# Qualifying a eukaryotic cell-free system for fluorescence based GPCR analyses

**DOI:** 10.1038/s41598-017-03955-8

**Published:** 2017-06-16

**Authors:** Anne Zemella, Solveig Grossmann, Rita Sachse, Andrei Sonnabend, Michael Schaefer, Stefan Kubick

**Affiliations:** 1 Fraunhofer Institute for Cell Therapy and Immunology (IZI), Branch Bioanalysis and Bioprocesses, Potsdam-Golm, Am Mühlenberg 13, 14476 Potsdam, Germany; 2 Rudolf-Boehm-Institut für Pharmakologie und Toxikologie, Medizinische Fakultät, Härtelstraße 16-18, 04107 Leipzig, Germany

## Abstract

Membrane proteins are key elements in cell-mediated processes. In particular, G protein-coupled receptors (GPCRs) have attracted increasing interest since they affect cellular signaling. Furthermore, mutations in GPCRs can cause acquired and inheritable diseases. Up to date, there still exist a number of GPCRs that has not been structurally and functionally analyzed due to difficulties in cell-based membrane protein production. A promising approach for membrane protein synthesis and analysis has emerged during the last years and is known as cell-free protein synthesis (CFPS). Here, we describe a simply portable method to synthesize GPCRs and analyze their ligand-binding properties without the requirement of additional supplements such as liposomes or nanodiscs. This method is based on eukaryotic cell lysates containing translocationally active endogenous endoplasmic reticulum-derived microsomes where the insertion of GPCRs into biologically active membranes is supported. In this study we present CFPS in combination with fast fluorescence-based screening methods to determine the localization, orientation and ligand-binding properties of the endothelin B (ET-B) receptor upon expression in an insect-based cell-free system. To determine the functionality of the cell-free synthesized ET-B receptor, we analyzed the binding of its ligand endothelin-1 (ET-1) in a qualitative fluorescence-based assay and in a quantitative radioligand binding assay.

## Introduction

G protein-coupled receptors (GPCRs) are known to form the largest class of transmembrane proteins in humans. Currently more than 1000 annotated members of GPCRs are characterized by a general topology composed of seven transmembrane-spanning helices (TM1-7) connected by three extracellular (E1-3) and three intracellular (C1-3) loops. GPCRs are key elements regulating intracellular signaling cascades activated by extracellular stimuli such as odorants, light, peptides, neurotransmitters and hormones. Furthermore, GPCRs serve as targets for more than 50% of all modern pharmaceutical drugs^1^. As a consequence, the characterization of GPCRs has become a research topic of enormous economic importance. So far, over 100 GPCR structures such as bovine rhodopsin receptor^2^, β_2_-adrenergic receptor^3^ and CXCR4 chemokine receptor^4^ could be crystallized and solved at atomic resolution. Recently the crystal structure of the ET-B receptor was solved as well^5^. For these receptors a structural-based virtual ligand screening is possible, that models new derivatives of known ligands to improve its characteristics. For GPCRs with an unknown crystal structure time-consuming and cost-intensive high-throughput screenings of small compounds are commonly performed *in vivo*. Therefore, novel screening methods as an alternative to common ligand discovery strategies are urgently desired.

As an alternative, cell-free protein synthesis (CFPS) has gained increasing interest to *in vivo* based expression systems^6^. In principle cell-free systems circumvent the effects of cellular signal cascades that might be activated by mistake by an overexpression of membrane proteins in living cells. Cell-free protein synthesis is therefore a suitable tool for the efficient production of GPCRs. In the last years several reports delineating cell-free synthesis of membrane proteins mainly in *E. coli* and wheat germ but also in insect and Chinese ovary hamster cell lysates were published^7–12^. *E. coli* and wheat germ systems in particular exhibit an enormous productivity leading to several mg/ml of de novo synthesized protein. Nevertheless the absence of membrane organelles in these systems often results in precipitated protein. Solubilization of membrane proteins is therefore achieved by the addition of either lipids in the form of nanodiscs, liposomes or detergents. According to this methodology the successful synthesis of a broad range of GPCRs in cell-free systems was achieved^7^. Depending on further downstream applications the choice of the membrane topology is essential. Planar membranes in the form of nanodiscs are often preferable for ligand binding studies since both sides of the statistically integrated GPCR are always accessible to the ligand. In contrast a site-directed integration of GPCRs into membrane structures might be desired for certain applications, such as the identification of epitope specific antibodies directed against specific subdomains of a membrane protein^7^. In principle various vesicular membranes such as liposomes and microsomes are valuable tools to address this question. Nevertheless liposomes exhibit a bidirectional, passive integration of membrane proteins into the lipid bilayer due to the missing ribosome-translocon complex. In a current report based on an *E. coli* cell-free system, parts of the Sec translocon machinery were reconstituted inside of cell-sized liposomes during the synthesis of the target protein^8^. With this modification the productivity of the system was significantly increased. Moreover the membrane integration of the produced proteins was improved. Therefore the presence of a functional translocon complex might be a notable advantage for membrane protein synthesis and analysis in eukaryotic cell-free systems harboring endogenous microsomal structures.

Eukaryotic lysates from cultured insect (Sf21) and Chinese hamster ovary cells (CHO) contain endogenous microsomal structures with an integrated natural translocon^13, 14^. The productivity of these systems is currently lower in comparison to *E. coli* and wheat germ systems. Nevertheless the presence of a natural translocon might lead to a high translocation efficiency resulting in correctly folded and membrane embedded proteins, which is a prerequisite for the synthesis of functionally active proteins.

In this study, we have chosen the well-known ET-B receptor as model protein for the cell-free synthesis in an insect based cell-free system. The class A GPCR induces vasodilation after recognition of the ET-1 peptide^15^. We show the successful CFPS of ET-B in combination with time-saving fluorescence-based methods to characterize localization, integration and orientation in endogenous microsomes as well as its ligand-binding properties as a basis for further development of cell-free-based screening assays. A quantitative measurement of ligand affinity revealed a typical ET-1 binding curve, thereby showing the correct binding parameters of the cell-free produced ET-B receptor.

## Results

### Localization studies of the transmembrane protein ET-B

To illustrate the function of GPCRs synthesized in an insect-based cell-free system, the Endothelin B receptor was chosen as a model protein. In particular, the first part of this report is focused on the localization, integration and orientation of synthesized ET-B receptor into microsomal membranes since its correct folding and functionality requires a proper membrane insertion^16^. Translation and transcription reactions were performed in batch mode in the absence of any detergents. Calculated protein yields were 8 µg/ml and, thus, in the range of previously reported amounts of cell-free synthesized proteins^17^. In addition to the potential to form posttranslational modifications, microsomal structures in insect cell lysate enable a localization of proteins in their membrane structure as well as the translocation of proteins into their lumen^18^.

**Figure 1:**
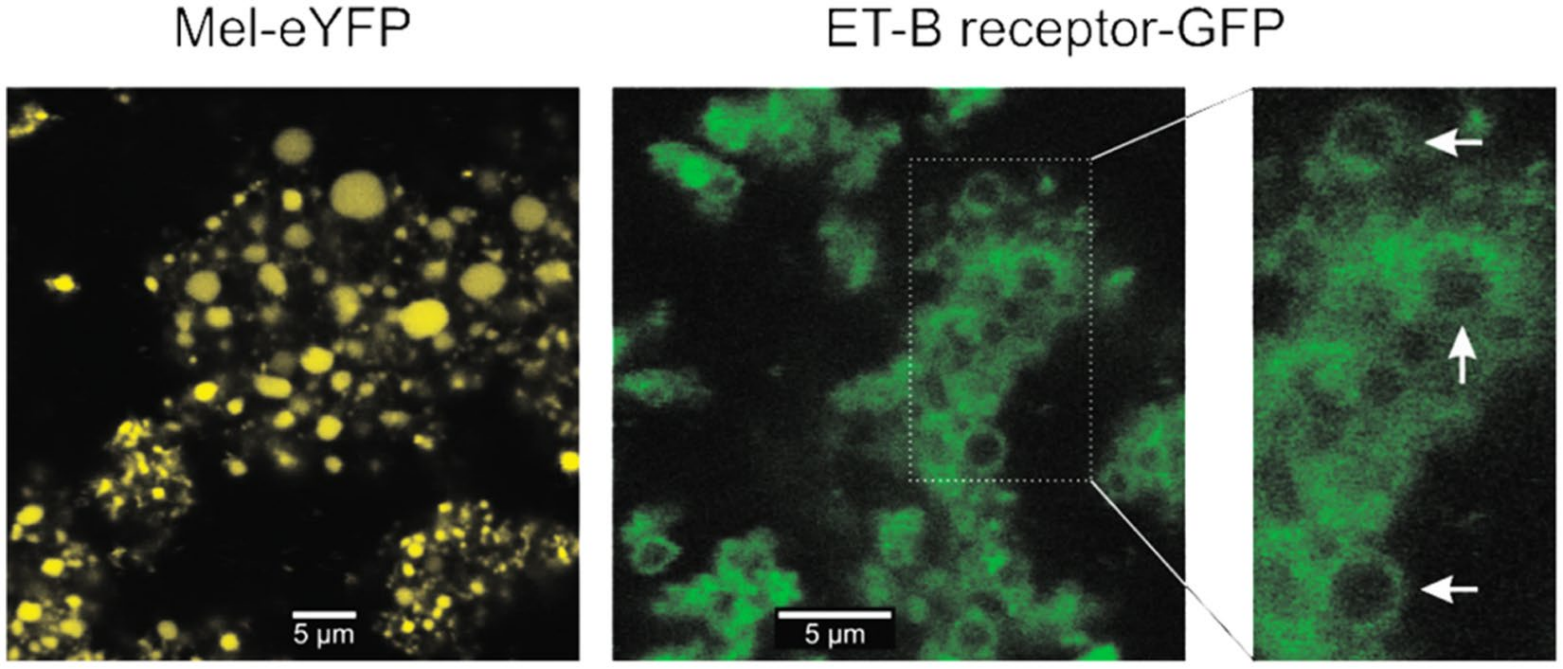
Comparison of the luminal versus transmembrane topology of *in vitro* translated Mel-eYFP and ET-B-GFP in insect cell extracts. Analysis was performed by confocal laser scanning microscopy after allowing vesicular structures to swell for 15 min under hypoosmotic conditions in PBS. Mel-YFP fluorescence (left panel) was evenly distributed within larger vesicles, indicating intraluminal localization. By contrast, ET-B-GFP fluorescence (right panels) was enhanced in vesicle membranes (see arrows in inset with higher magnification), suggesting a transmembrane topology of the protein.

In order to distinguish between a translocation into the microsomal lumen and the localization in microsomal membranes, two fluorescent fusion proteins were constructed. First, the cytosolic eYFP was coupled to the well-characterized signal peptide sequence of honeybee melittin (ref. 19; Mel-eYFP) to demonstrate a translocation into the microsomal lumen^20^. Second, GFP was fused to the membrane protein C-terminus of ET-B receptor to determine the localization in microsomal membranes (Fig. 1).

CLSM data received for Mel-eYFP synthesized in standard insect lysate indicates that the fusion protein was translocated into the microsomal lumen as shown by a defined fluorescent signal. In contrast to the equally distributed fluorescence signal of Mel-eYFP, the transmembrane ET-B receptor showed a clear signal in microsomal membranes well illustrated by the enlarged picture in Fig. 1. The observed diameters for microsomal structures (1–1.5 µm) are in accordance with previously recorded confocal images^21^. In addition the localization of cell-free synthesized membrane proteins with respect to the microsomal structures was analyzed by using an ER-Tracker. The fluorescence signal of ET-B-eYFP clearly co-localized with the signal of the stained microsomes. This co-localization visualized the successful translocation of the secretory model protein Mel-eYFP into the microsomal lumen and the integration of transmembrane protein ET-B-eYFP into the microsomal membranes (Supplementary Figure S1).

### Integration and orientation studies of the transmembrane protein ET-B

**Figure 2:**
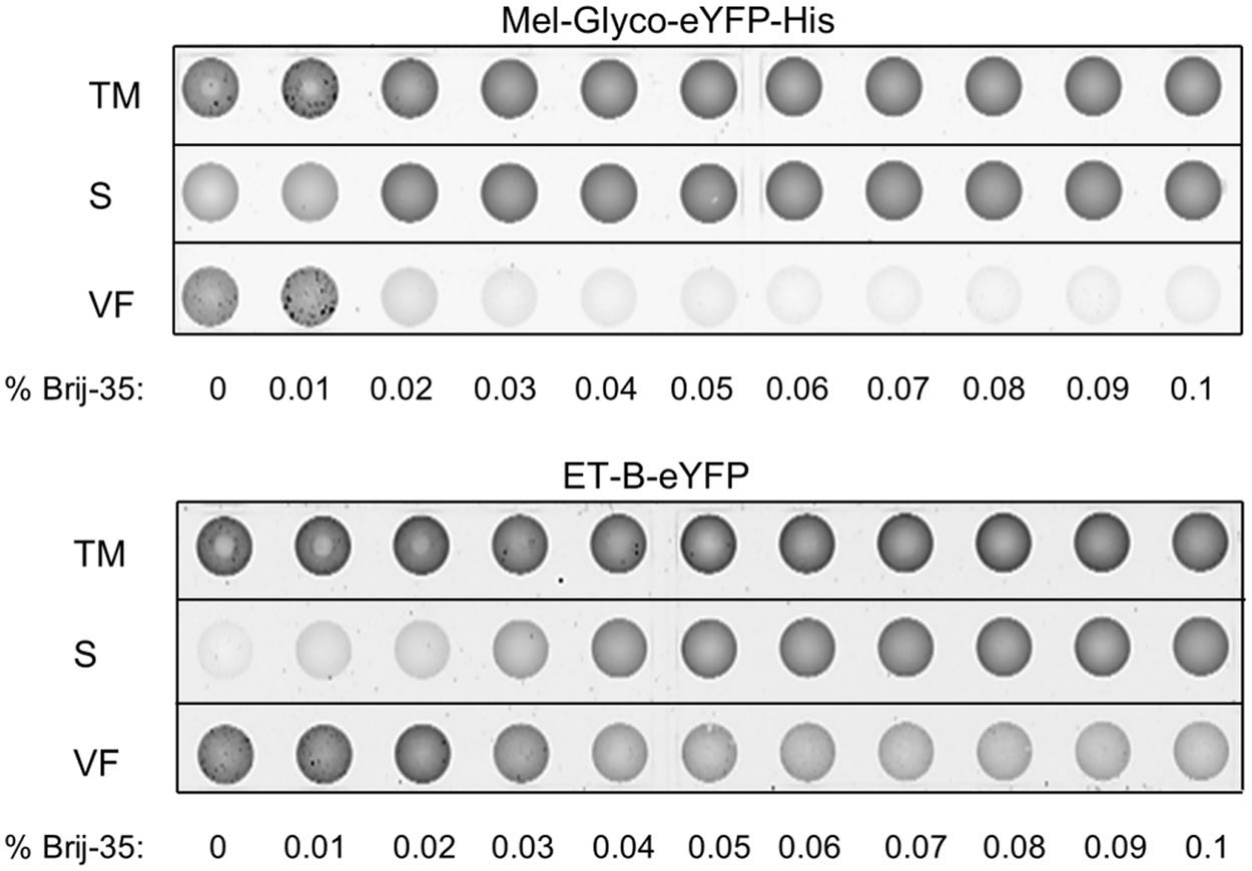
Localization of cell-free synthesized secreted and transmembrane proteins. Upper panel: The localization of the secreted protein Mel-Glyco-eYFP-His was determined by fluorescence analysis in the presence of defined Brij35 concentrations. The amount of Mel-Glyco-eYFP in the supernatant fraction indicates the state of perforation of microsomal membranes. Lower panel Fluorescence analysis of the localization of the transmembrane protein ET-B-eYFP in the presence of defined Brij35 concentrations. By applying higher Brij35 concentrations, the transmembrane protein ET-B-eYFP is slowly transferred into the supernatant.

The precise integration of the ET-B receptor into biological membranes is a basic prerequisite for subsequent functional characterization and, therefore, has been analyzed in detail. A perforation procedure of microsomal vesicles can be used to determine the release of translocated versus membrane integrated proteins into the supernatant. Therefore, the influence of different Brij35 concentrations was analyzed. The localization of the cytosolic protein Mel-Glyco-eYFP-His as well as the membrane protein ET-B-eYFP was determined in the presence and absence of Brij35. After an initial centrifugation step, the translation mixture (TM) was subdivided into the vesicular fraction (VF) and a supernatant fraction. The microsomes-containing fraction (VF) was resuspended in PBS supplemented with defined Brij35 concentrations. After a second centrifugation step, the supernatant (S) and vesicular fraction (VF) were collected and applied to Ibidi-slides. Finally, the localization of eYFP-labeled protein was visualized by fluorescence measurement using a phosphorimager (Typhoon TRIO + Imager, GE Healthcare; Fig. 2).

For the determination of the amount of released protein, fluorescence signals of the tagged proteins in the translation mixture, in supernatant and in the vesicular fraction were analyzed (Supplementary Table S2). In the absence of Brij35, a bright fluorescent signal was obtained in the TM and VF, whereas only a minor fluorescence signal was detected in the supernatant (Fig. 2). The fluorescence signal, visualized in the vesicular fraction, indicates a luminal location of Mel-Glyco-eYFP-His. The same result was obtained in the presence of 0.01% Brij35. Upon addition of 0.02% Brij35 the distribution of fluorescent proteins changed. Almost the entire fluorescence signal of the 33 kDa protein Mel-Glyco-eYFP-His was detected in the supernatant, indicating the protein’s release into the supernatant and thus a successful perforation of vesicular structures. At higher Brij35 concentrations (above 0.03%) maximum fluorescence signals were observed in the TM and supernatant fraction, indicating the complete release of Mel-Glyco-eYFP into the supernatant.

Different results were obtained for the ET-B receptor. In the absence of Brij35, a weak fluorescent signal was detected in the supernatant and a bright fluorescence signal was visualized in the TM and VF. In contrast to Mel-Glyco-eYFP-His, where the addition of 0.02% Brij35 resulted in a perforation of the microsomal membrane and a nearly complete release of the protein, only a faint fluorescence signal of ET-B-eYFP in the supernatant could be observed at this Brij35 concentration. Upon rising the concentration of Brij35 above 0.03%, only a slowly emerging fluorescence signal in the supernatant was detected. Even at higher Brij35 concentrations (above 0.06%) a fluorescence of the eYFP-tagged protein was still observed in the VF. These findings indicate that the majority of cell-free synthesized ET-B receptors are integrated into microsomal membranes.

**Figure 3:**
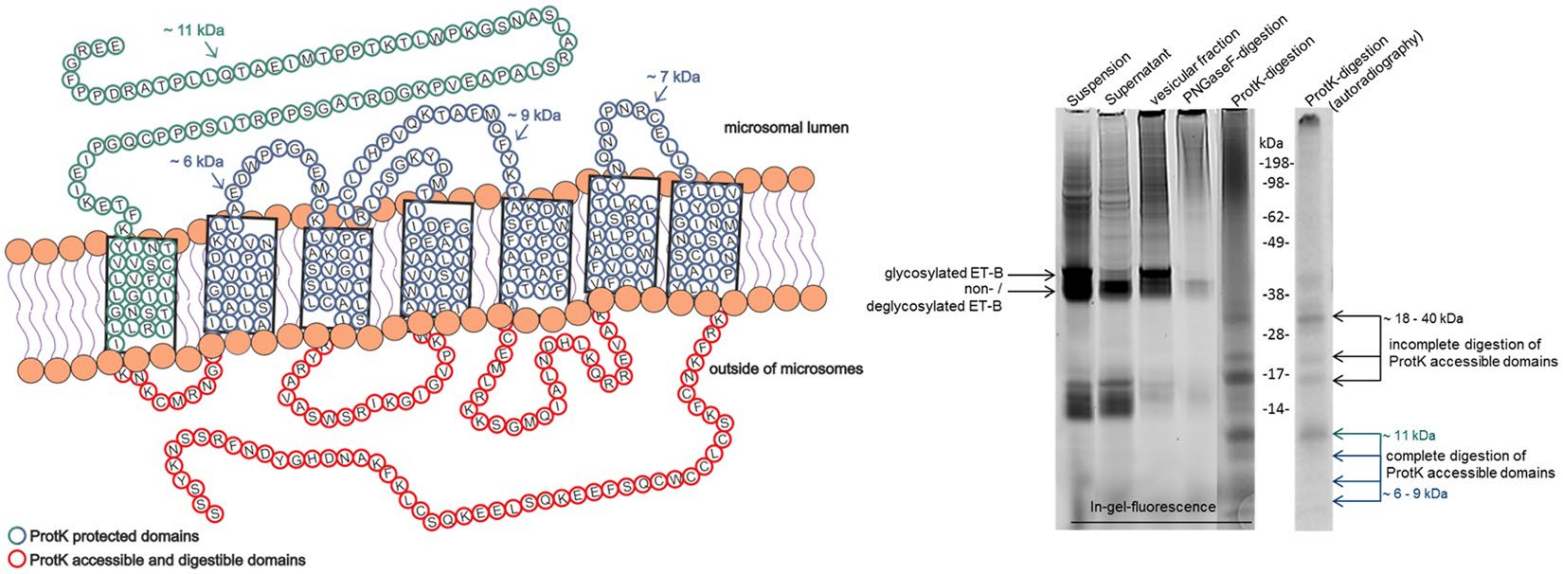
Schematic illustration of the ET-B receptor embedded in microsomal membranes and comparison of in-gel-fluorescence and autoradiographic analysis of the synthesized ET-B receptor. Left panel: Shown in red are proteinase K accessible domains that will probably be digested by the enzyme. Proteinase K protected domains are colored in green and blue. Right panel: ET-B was synthesized in the presence of a precharged tRNA harbouring the fluorescence dye Bodipy, coupled to lysine. Bodipy was used to visualize protein bands as alternative to radioactively labeled leucine bands. PNGaseF and ProtK-digestion were performed with the vesicular fraction. Green and blue arrows indicate the corresponding bands to the illustrated domains in the left panel. Black arrows indicate upper bands that might result of incompletely digested domains outside of the microsomal membrane.

Complementary to the radiolabeled protein analysis we probed a precharged tRNA harboring the fluorescent dye Bodipy, coupled to lysine in our cell-free mixture. The fluorescent lysine analog is co-translationally incorporated into the synthesized ET-B receptor. In-gel fluorescence analysis revealed a defined band, corresponding to the full-length ET-B receptor with a calculated molecular weight of 49 kDa (Fig. 3, right panel). According to the hydrophobic transmembrane domains of membrane proteins and the following interaction with SDS, a lower apparent molecular weight can be detected^22^. Moreover, a protein band showing a higher molecular weight was detected in the suspension as well as in the vesicular fraction. After PNGaseF digestion this band particularly disappeared, indicating a glycosylation of the cell-free-produced ET-B receptor. The presence of glycosylated receptor protein indicates the translocation of the ET-B receptor into the ER-derived microsomal membranes. The lower band showing a molecular weight of 17 kDa corresponds to the excess of fluorescently labeled tRNA coupled to lysine. To further elucidate the integration of the radiolabeled ET-B receptor in microsomal membranes, the proteins in the vesicular fraction were digested using proteinase K. As a result, a defined band pattern of radiolabeled proteins was detected in the in-gel-fluorescence as well as in the autoradiograph (marked by arrows). Whereas only slight bands were detected by autoradiography, the in-gel-fluorescence provides a highly sensitive detection method. The defined band pattern indicates that only certain extra vesicular receptor domains were exposed and accessible for ProtK digestion (Fig. 3; left panel). Assuming a complete digestion of ProtK accessible domains of ET-B receptor we expect four peptide fragments in the range of 6–11 kDa. These bands can be clearly detected using in-gel fluorescence whereas the autoradiography only shows a band at an estimated molecular weight of 11 kDa. Additional upper bands might occur due to an incomplete digestion of ProtK accessible domains the of ET-B receptor. Assuming that only certain extra vesicular domains were digested by ProtK we expect peptide fragments in the range of 18–40 kDa. These expectations were confirmed by in gel-fluorescence as well as autoradiography. Our results support the suggestion of a correctly integrated ET-B receptor in microsomal membranes.

### Binding studies of a specific ET-1 ligand to the transmembrane protein ET-B

**Figure 4:**
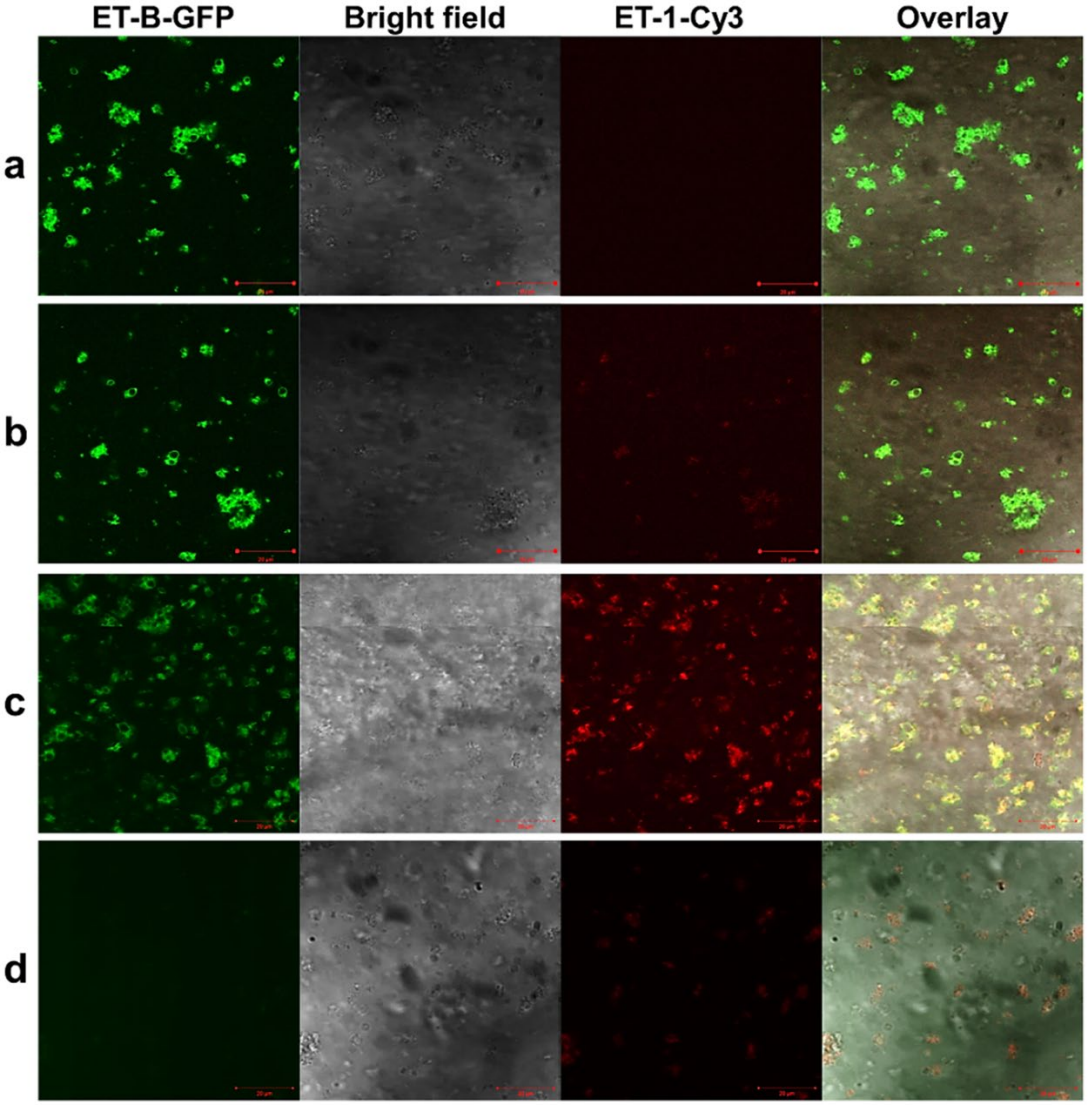
Determination of ligand binding using confocal laser scanning microscopy. (**a**) Confocal imaging of ET-B-GFP in absence of any ligands; (**b**) imaging of ET-B-GFP and ET-1-Cy3 in absence of any detergents; (**c**) Co-localization of ET-B-GFP and Cy3-ET-1 in the presence of 0.03% Brij35; (**d**) imaging of negative control and Cy3-ET-1 in the presence of 0.03% Brij35.

All experiments were performed on a cover slip, using CLSM as a fast and sensitive optical read out. The vesicular fraction containing microsomes with integrated ET-B-GFP was applied to cover slips and analyzed. Specific binding of the ET-1 ligand was determined in the presence (0.03%) and absence of Brij35. First, the localization of synthesized ET-B-GFP was analyzed in the absence of any ET-1-Cy3 ligand (Fig. 4 line a). As described before, the overlay of brightfield images and ET-B-GFP fluorescence clearly showed a maximal intensity of GFP-tagged protein in microsomal membranes, indicating a successful integration into vesicular membranes.

In the second part (Fig. 4b) the vesicular fraction was incubated with the Cy3 labeled ligand ET-1 in the absence of any detergent. Again the maximum intensity of GFP-tagged protein was recorded in microsomal membranes. In addition, a faint fluorescent signal was detected for the ET-1-Cy3 ligand in microsomal membranes. Nevertheless, the overlay of ET-B-GFP, brightfield and ET-1-Cy3 mainly displays the co-localization of the fluorescently tagged protein and microsomal membranes whereas the faint fluorescence signal of ET-1-Cy3 could not be clearly detected.

In Fig. 4c the vesicular fraction was again incubated with Cy3-labeled ET-1 in the presence of 0.03% Brij35. This concentration was chosen according to the perforation analysis (see Fig. 2). In the presence of 0.03% Brij35 the vesicular membrane is perforated to an extent that small molecules were able to pass the membrane whereas membrane proteins mostly remain still embedded in the vesicular membrane. In contrast to the previously described results (compare Fig. 4b), a bright fluorescence of the ET-1-Cy3 ligand was obtained in the microsomal fraction. The overlay clearly shows a co-localization of ET-1-Cy3 and its receptor protein ET-B-GFP, indicating a successful binding of the ET-B ligand (Fig. 4c). Figure 4d shows the low non-specific binding of ET-1-Cy3 in the absence of ET-B-GFP. This experiment was performed in presence of 0.03% Brij35 to exclude a diffusion effect.

**Figure 5:**
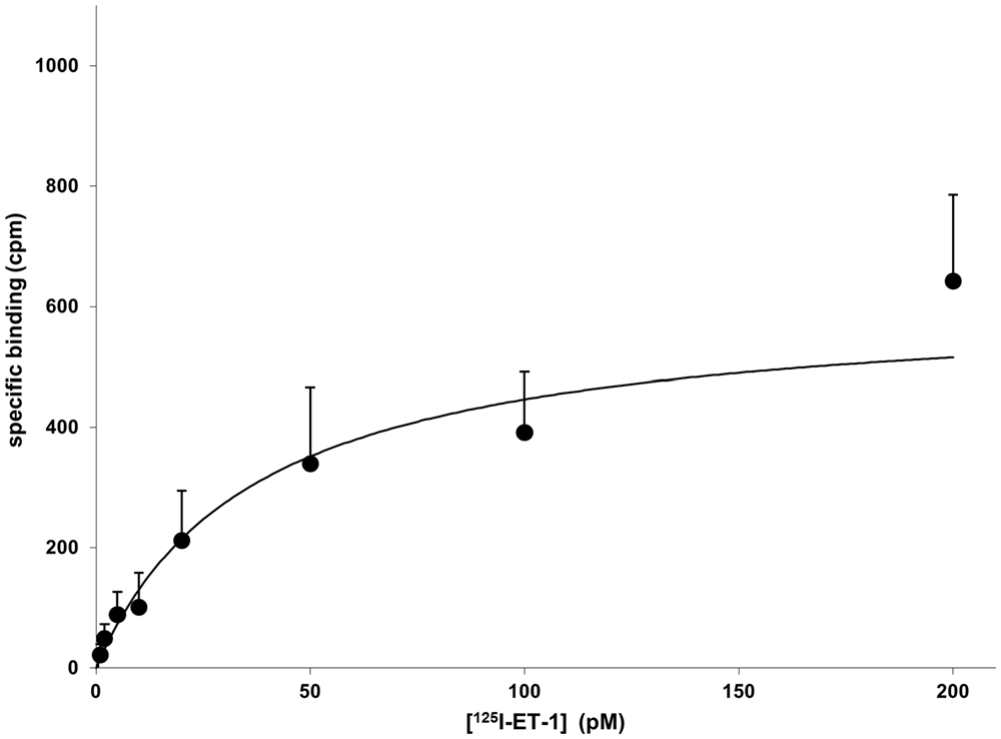
Radioligand binding assay of radiolabeled ^125^I-ET-1 to ET-B-GFP. 8 ng of cell-free synthesized ET-B-GFP was incubated with increasing concentration of ^125^I-ET-1. ^125^I-ET-1 binding was analyzed in the presence of 0.03% Brij35. As negative control (NTC) vesicular fraction without cell-free synthesized ET-B was used.

To validate the ET-B ligand binding, a radioligand binding assay was performed with the vesicular fraction containing 8 µg/ml ET-B. In this context, the affinity value K_D_ and the maximum density of receptors B_max_ was determined using ^125^I-ET-1 as a ligand. To this end the vesicular fraction was used without further purification steps. The applied concentration of radioligand ^125^I-ET-1 with constant specific activity was increased ranging from 0 pM up to 200 pM until a saturating effect was observed (Fig. 5; Supplementary Table S3). For the ET-B receptor synthesized in a cell-free insect based system, a typical binding curve was received with a K_D_ value of 36.8 ± 13.1 pM (t = 2.8, p = 0.0265) and a B_max_ of 610.3 ± 131.5 cpm (corresponds to 53.7 pmol/mg). B_max_ value was obtained from the illustrated saturation plateau. The dissociation constant K_D_ was calculated as half of the B_max_ value. The gained results indicate a high affinity of ET-1 binding to the receptor ligands. To determine the active fraction of ET-B receptor we assumed a 1:1 binding model. Including the molecular weight of ET-B-GFP (75 kDa) and the calculated B_max_ value of 53.7 pmol/mg an active receptor fraction of 0.40% was determined.

## Discussion

G protein-coupled receptors perceive many extracellular signals and their central role in activating and regulating intracellular signal cascades makes them a promising target for drug discovery. Currently, more than 100 orphan GPCRs with unidentified ligands still exist^23^. Several strategies were developed until now to facilitate and improve the screening and identification of possible ligands. These strategies include crystal structure-based drug design, high-throughput screening (HTS) and sensor-based ligand screening methods. As novel alternative to commonly used screening methods, we present a ligand-binding approach based on a cell-free protein synthesis system offering a time-saving, optical read-out-based and highly parallel processable method. Identification of novel ligands and small molecules is commonly performed *in vivo* by using HTS methods^24^. Thereby a large amount of different compounds is applied to GPCR-overexpressing cells and afterwards the second messenger and effector response is evaluated. Such cell-based systems are complex and therefore contain several drawbacks: Besides the time-consuming and cost-intensive cultivation and preparation of cells, the overexpressed GPCR might undergo cell-specific processing, including internalization and potential interaction with endogenous activators resulting in over-stimulated signal cascades and cytotoxic side effects^25^. The presented cell-free system circumvents effects such as internalization and non-specific activation of second messenger cascades, thereby enabling a successful ligand screening of difficult-to-express GPCRs. To qualify cell-free protein synthesis systems as high-throughput screening methods, comparable ligand binding affinities of the target GPCR as determined in cell-based systems are essential. For this reason we have chosen the well-studied ET-B receptor with known binding kinetics as a model protein. To evaluate the optical confocal laser scanning microscopy based read-out of the ET-B bound and fluorescently labeled ET-1-Cy3, a radioligand binding assay was performed in parallel. The obtained affinity value of 28.6 pM is in good agreement with the *in vivo* determined dissociation constant in human tissues (tissue specific dissociation constant is usually in the range of 10 to 150 pM; ref. 26). After perforation of ER-derived microsomal membranes a defined fluorescence signal displays the binding of Cy3-ET-1 to its receptor. These results indicate that the ET-1 ligand binding site is positioned in the lumen of microsomes. Accordingly the recently published activation mechanism of endothelin ET-B receptor by endothelin-1 identified the extracellular portion of the receptor as ligand binding domain^5^. Moreover this observation is in good accordance with the results of Klammt *et al*. confining a 39 amino acid area between P93 (N-terminal domain) and C131 (TM1) as ET-1 binding site^27^.

In the mentioned studies the radioligand and fluorescence binding assay was performed in the presence of detergents (1% Brij78; 1% DDM and 0.03% Brij35), to gain access to the luminally orientated ligand binding site upon correct translocation of the GPCR into ER-derived microsomes. However, the presence of detergent may result in altered ligand-binding affinities and efficiencies^28^. In addition, the perforation assay indicates that approximately 40% of ET-B receptor molecules were determined in the supernatant fraction when using 0.03% Brij35. Some of these molecules are therefore presumably not membrane-integrated and display probably a changed conformation that might lead to a loss of ligand binding ability. Additionally the presence of small microsomes that can not be pellet at 16.000 × g might lead to membrane embedded and unprecipitated functional receptor protein. A hint for this assumption can be seen in the faint glycosylation band in the supernatant fraction. This band should only occur upon correct protein translocation into the lumen of microsomes followed by protein glycosylation. Assuming 60% of our synthesized receptor being membrane integrated, only 0.5% of the ET-B receptors display ligand binding activity. This result indicates that the fluorescently tagged membrane protein indeed serves as successful protein translation control but not as a control for correctly folded protein. In comparison to previously reported studies, where a total amount of 20.9 ng/ml^24^ active ET-B receptor was synthesized, 32 ng/ml were obtained in our study. In our study the microsomal fraction was directly used for binding analyses. An enriched amount of ET-B receptor is expected in this particular fraction. In comparison to a purified receptor expressed in baculovirus-infected insect cells with a determined binding activity of 19.6 nmol/mg^29^, the binding activity of cell-free synthesized receptor was in the range of 53.7 pmol/mg, displaying a substantially lower binding activity. The estimated fraction of active receptor (<0.5%) in the described eukaryotic cell-free protein synthesis system might be significantly improved in the near future. In particular since previously performed studies addressing cell-free systems of eukaryotic origin also obtained an active fraction of ET-B below one percent^17^. According to Merk *et al*. insertion and translocation efficiency has been identified as main limiting factor in this particular cell-free system. Several groups currently address the bottleneck of translocation efficiency *in vivo* ^30, 31^. Pechmann *et al*. declare a cluster of non-optimal codons downstream of the signal recognition particle (SRP)-binding site as enhancer for nascent-chain connecting to SRP. A second finding is based on the sequence approximately 100 residues downstream of the transmembrane domain. The results show that conformational effects of the downstream sequence influence the membrane integration mechanism^30^. Moreover the addition of further membrane protein specific chaperones to improve folding and a translocation process might increase the receptor’s activity. In particular, a detailed analysis of cell-free extracts is needed to determine the factors and auxiliary components for membrane translocation and insertion^32^. Interestingly, the analysis of the binding affinity resulted in a comparable value to the constants determined *in vivo*, although the ET-B receptor was inserted in an ER-derived membrane in the cell-free system instead of a plasma membrane. This is of highest interest since the lipid composition can strongly influence a GPCR’s activity and stability^33^. For this reason sensor-based ligand screening that usually deals with the immobilization of purified GPCRs on surface plasmon resonance (SPR) surfaces nowadays implements novel strategies to embed GPCRs in more natural environments. These environments include nanodiscs with a defined lipid-composition and lipid-vesicles^28^. Furthermore, an indirect immobilization strategy of GPCRs through nanodiscs and lipid-associated tags is desirable to analyze the binding affinities of ligands to GPCRs. Nevertheless, an automation process for the high-throughput analysis of cell-free produced GPCRs is still missing. The direct integration of cell-free synthesized GPCRs in vesicular structures and their subsequent optical analysis might be the link to establish an automation process for high-throughput sensor-based screenings in this context. The incorporation of biotinylated lipids into vesicles to immobilize GPCR-containing microsomes on streptavidin-coated surfaces was already shown^34^. Demonstrating in genera the feasibility of this approach, cell-free protein synthesis is predestined for high-throughput analysis since protein synthesis reactions can be automatically performed by adding different DNA/mRNA templates to the cell-free reaction in a highly parallel procedure^35^. In addition, the small initial screening volumes in the microliter range can be linearly scaled up to larger volumes for subsequent production processes. Moreover, the efficient downstream analysis by optical amplification presents a novel technique combining cell-free on-chip-protein synthesis with sophisticated ligand screening approaches. Until now various automated and chip-based applications of cell-free protein synthesis in microsystems have been established^26, 36, 37^. In the near future further development of fluorescence based methods in cell-free systems by incorporating non-canonical amino acids into membrane proteins at desired positions followed by their conjugation with fluorescent dyes might offer additional alternatives for functional protein analysis^38, 39^. In this study initial experiments using fluorescence based methods were performed to determine the membrane integration of cell-free synthesized ET-B using proteinase K digestion in combination with in-gel-fluorescence analysis. These experiments were followed by a ligand-binding analysis using CLSM. Up to date an emerging number of fluorescence and in particular bioluminescence resonance energy transfer-based methods are used to study GPCR interaction and activation in cell cultures^40^. These assays usually enable high resolutions in combination with highly sensitive fluorescence detection of conformational changes after ligand-binding^41^. The extension of the presented methods to cell-free systems enables novel perspectives for future applications.

## Materials and Methods

### DNA template design

To determine the fluorescence signal of translocated and membrane integrated proteins the following samples were selected: (1) ET-B-eYFP and (2) ET-B-GFP: ET-B receptor C-terminally fused to eYFP or GFP; (3) Mel-Glyco-eYFP-His: eYFP construct fused to Melittin (Mel) signal sequence with introduced glycosylation site (-N-X-S/T-) and His affinity tag; (4) Mel-eYFP: eYFP fusion construct harboring a Mel signal sequence to study translocation efficiency. Linear DNA templates were generated by two-step Expression-PCR (E-PCR; ref. 42). In the first step, gene-specific primers were used to amplify coding sequences of ET-B-eYFP, ET-B-GFP, Mel-Glyco-eYFP-His or Mel-eYFP from its *in vivo* (peGFP-N1-ET-B-GFP) and *in vitro* expression vectors (pIX3.0-Mel-Glyco-eYFP-His; pIX4.0-eYFP, pIX3.0-ET-B) respectively. In the case of ET-B, an overlapping sequence to eYFP was added at the 3′end of the ET-B encoding sequence. The following primers were used for the first PCR step: x-ETB-F, x-Mel-EXFP-F, ET-B-oe-eXFP-R and x-EXFP-R (Supplementary Table S1). In the second PCR step, regulatory sequences essential for cell-free synthesis were added. ET-B constructs were amplified with no-tag sense adapter primer (N-0 and C-0). The same adapter primers were used for Mel-eYFP. His-tag antisense primer (C-His) was used in case of Mel-Glyco-eYFP-His. In addition, a plasmid containing an internal ribosome entry site (IRES; ref. 14) upstream of the ET-B encoding sequence was manufactured by GeneArt (ThermoFisher Scientific). Subsequently a melittin signal sequence was added to this ET-B-encoding gene by PCR using the PCR primer oe-Mel-ETB-F.

### Cell-free protein synthesis

Cell-free protein synthesis was performed in the linked and coupled mode. In the linked mode, transcription and translation are separated by an intermediate gel filtration step. Transcription of E-PCR products was performed using T7 RNA-polymerase (Agilent, ref. 43) followed by RNA purification with DyeEx spin columns (Quiagen). Purified RNA (linked mode) or plasmid DNA (coupled mode) was afterwards added to the reaction mixture composed of 40% (v/v) insect lysate, 200 µM (linked mode) and 100 µM (coupled mode) amino acids and energy generation components. For the determination of protein yield and analysis via autoradiography, 23.08 dpm/pmol ^14^C-leucine was added. Protein synthesis was performed in batch mode for 180 min at 27 °C with gentle shaking at 500 rpm (Thermomixer comfort, Eppendorf, Hamburg, Germany). Cell-free synthesis was performed according to previously described protocols^44, 45^.

### Determination of protein yield

Aliquots of 5 µl were taken after 3 h reaction time, mixed with 3 ml trichloroacetic acid and incubated in 80 °C water bath for 15 min. After incubation on ice for 30 min, non-incorporated ^14^C-leucine was removed from the protein solution by a filtration step using a vacuum filtration system (Hoefer) as described previously^18^. Measurement of incorporated ^14^C-leucine was performed by liquid scintillation counting (LS6500 Multi-Purpose scintillation counter, Beckman Coulter).

### SDS-PAGE and in-gel fluorescence

8 µl of the translation mixture were subjected to cold acetone precipitation and centrifuged for 10 min at 16.000 ×  g at 4 °C. Pellets were dried for 1 h at 45 °C, resuspended in 20 µl of 1 × sample buffer (NuPAGE® LDC Sample Buffer, Invitrogen) and loaded on a precast SDS-PAGE gel (NuPAGE 10% Bis-Tris gel, Invitrogen). Gels were run for 35 min at 185 V and afterwards incubated in a water-methanol mixture (50% v/v) for 20 min. Bodipy-labeled proteins were excited at 532 nm and detected at 580 nm using a phosphorimager system (Typhoon TRIO + Imager, GE Healthcare). Deglycosylation of ET-B was performed using peptide-N-glycosidase F (PNGase F, NEB) as previously reported^21^. The protease protection assay using Proteinase K (NEB) was performed in a final volume of 10 µl. A 5 µl aliquot of the vesicular fraction was resuspended in PBS with 10 ng/µl Proteinase K. Incubation was carried out for 30 min on ice. The reaction was stopped with 6.25 mM phenylmethylsulfonyl fluoride (PMSF), followed by immediate acetone precipitation.

### Perforation of vesicles

Translation mixtures (TM) of synthesized Mel-Glyco-eYFP-His, ET-B-GFP and ET-B-eYFP were centrifuged at 16.000 × g for 10 min at 4 °C. The pellets were resuspended in PBS (phosphate-buffered saline, PBS Dulbecco without Ca^2+^ and Mg^2+^, Biochrom AG) and treated for 10 min with 0–0.1% polyoxyethylen(23)laurylether (Brij 35; Sigma Aldrich). To analyze the perforation of the microsomal membranes, the resuspended and detergent-treated pellet was centrifuged again at 16.000  ×  g for 10 min at 4 °C and divided into supernatant (S) and vesicular fraction (VF). Translation mixture, supernatant and vesicular fraction were applied to microwell glass slides (µ-Slide, 18 well, Ibidi) and cell-free synthesized eYFP-labeled proteins were excited at 488 nm. Fluorescence signals were recorded at 526 nm using a phosphorimager system (Typhoon TRIO + Imager, GE Healthcare).

### Microsome staining

Staining solution was prepared according to the manufacturer’s instructions. For microsome staining the endoplasmatic reticulum-Tracker (ER-Tracker™ Red, (BODIPY® TR Glibenclamide), Life Technologies) was diluted 1:1000 in PBS. Following to cell-free protein synthesis, target protein containing microsomes were harvested from 15 µl translation reactions by centrifugation (10 min, 16.000 × g, 4 °C) and washed three times with PBS. Washed microsomes were resuspended in 100 µl of the fresh staining solution. Staining was performed for 30 min at 27 °C while gentle shaking (500 rpm). Stained microsomes were centrifuged and washed again three times with 100 µl PBS. After washing, microsomes were resuspended in 15 µl PBS. The sample was diluted 1:5 and applied for microscopic examinations on microwell slides (µ-slide 18 well-flat, uncoated, Ibidi GmbH).

### Confocal laser scanning microscopy of microsomal vesicles

ER-Tracker-stained microsomes harboring cell-free synthesized eYFP fusion proteins were visualized by confocal laser scanning microscopy (CLSM). The laser scanning microscope unit (LSM 510, Carl Zeiss Microscopy GmbH) was equipped with a Plan-Aprochromat 63x/1.4 oil objective and eYFP was exited with an argon laser at 488 nm. After passing a long-pass filter (LP 505), the emitted light was captured using a photomultiplier. For ER-Tracker detection, samples were excited with a HeNe laser at 543 nm and emission was detected in the range of 550 to 593 nm. Similar settings were used for the detection of the ET-B-GFP fusion construct (ex. 488 nm, em. above 505 nm) and cyanine-3-labeled ligands (cy3; ex. 543, em. 570). Hypoosmotic conditions were reached by decreasing the sodium chloride content in comparison to the used PBS.

### Radio ligand binding assay

50 µl translation mixture was centrifuged at 16.000 × g for 10 min and the microsomal fraction (VF) containing the target protein ET-B-GFP (8 µg/ml) was resuspended in 5 ml Tris-BAME (50 mM Tris, 2 mM EGTA, 10 mM MgCl_2_, 0.15 mM Bacitracine, 0.0015% Aprotinin) buffer. Vesicular structures were perforated with 0.03% Brij35. 100 µl of diluted vesicular fraction (corresponding to 8 ng ET-B receptor) were mixed with ^125^I-ET-1 (Amersham Biosciences, USA) in different concentrations ranging from 0 pM to 200 pM to determine total binding or in presence of 1 µM ET-1 (Sigma-Aldrich, Steinheim, Germany) to determine unspecific binding. Specific binding was calculated by subtracting unspecific binding from total binding. Mixtures were incubated for 2 h at 27 °C with gentle shaking at 500 rpm. Afterwards samples were transferred onto polyethylenimine-treated GF/C filters (Whatman, Germany) and washed with PBS using a Brandell cell harvester. Filters were dried and transferred into 5 ml vials. Radioactivity was determined by liquid scintillation counting. Ligand binding assay was performed until a saturation effect was observed. Based on the observed saturation curve the maximum density of receptors (B_max_) as well as the affinity constant was calculated. The standard deviation and confidence intervals were calculated by using Sigmaplot 13 (Systat Software, Erkrath, Germany) and Marquardt-Levenberg algorithm.

## Electronic supplementary material

Supplementary
