# Supplementary material for: Qualifying a eukaryotic cell-free system for fluorescence based GPCR analyses
Source: Sci Rep. 2017 Jun 16;7:3740. doi: 10.1038/s41598-017-03955-8 (PMC5473880; doi:10.1038/s41598-017-03955-8)
Supplement: Supplementary file 1 — Supplementary [file 41598_2017_3955_MOESM1_ESM.pdf]

Qualifying a eukaryotic cell-free system for fluorescence based GPCR analyses

Anne Zemella[1], Solveig Grossmann[2], Rita Sachse[1], Andrei Sonnabend[1], Michael Schaefer[2] and Stefan Kubick[1*]

Supplementary Table 1. **Oligonucleotide sequences used for DNA template design**

Name	Oligonucleotide sequence
**x-ETB-F**	5'CTT TAA GAA GGA GAT AAA CAA TGC AGC CGC CTC CAA GTC 3'
**x-Mel-eXFP-F**	5' TAC ATT TCT TAC ATC TAT GCG GAC GTG AGC AAG GGC GAG GAG C 3'
**ET-B-oe-eXFP-R**	5' CTTGCTCACCTCTAGACAAGATGAGCTGTATTTATTACT 3'
**x-eXFP-R**	5' CTT GGT TAG TTA GTT ATT ACT ACT TGT ACA GCT CGT CCA TGC CG 3'
**N-0**	5' -Biotin-ATG ATA TCT CGA GCG GCC GCT AGC TAA TAC GAC TCA CTA TAG GGA GAC CAC AAC GGT TTC CCT CTA GAA ATA ATT TTG TTT AAC TTT AAG AAG GAG ATA AAC AAT G- 3'
**C-0**	5' -TAA TAA CTA ACT AAC CAA GAT CTG TAC CCC TTG GGG CCT CTA AAC GGG TCT TGA GGG GTT TTT TGG ATC CGA ATT CAC CGG TGA TAT CAT-Biotin- 3'
**C-His**	5'-TGG GGT CAC CAC CAT CAC CAT CAT TAA TAA CTA ACT AAC CAA GAT CTG TAC CCC TTG GGG CCT CTA AAC GGG TCT TGA GGG GTT TTT TGG ATC CGA ATT CAC CGG TGA TAT CAT-Biotin- 3'
**Oe-Mel-ETB-F**	5'TCTTACATCTATGCGGACGAGGAACGCGGTTTCCC3'

Untranslated regions are underlined

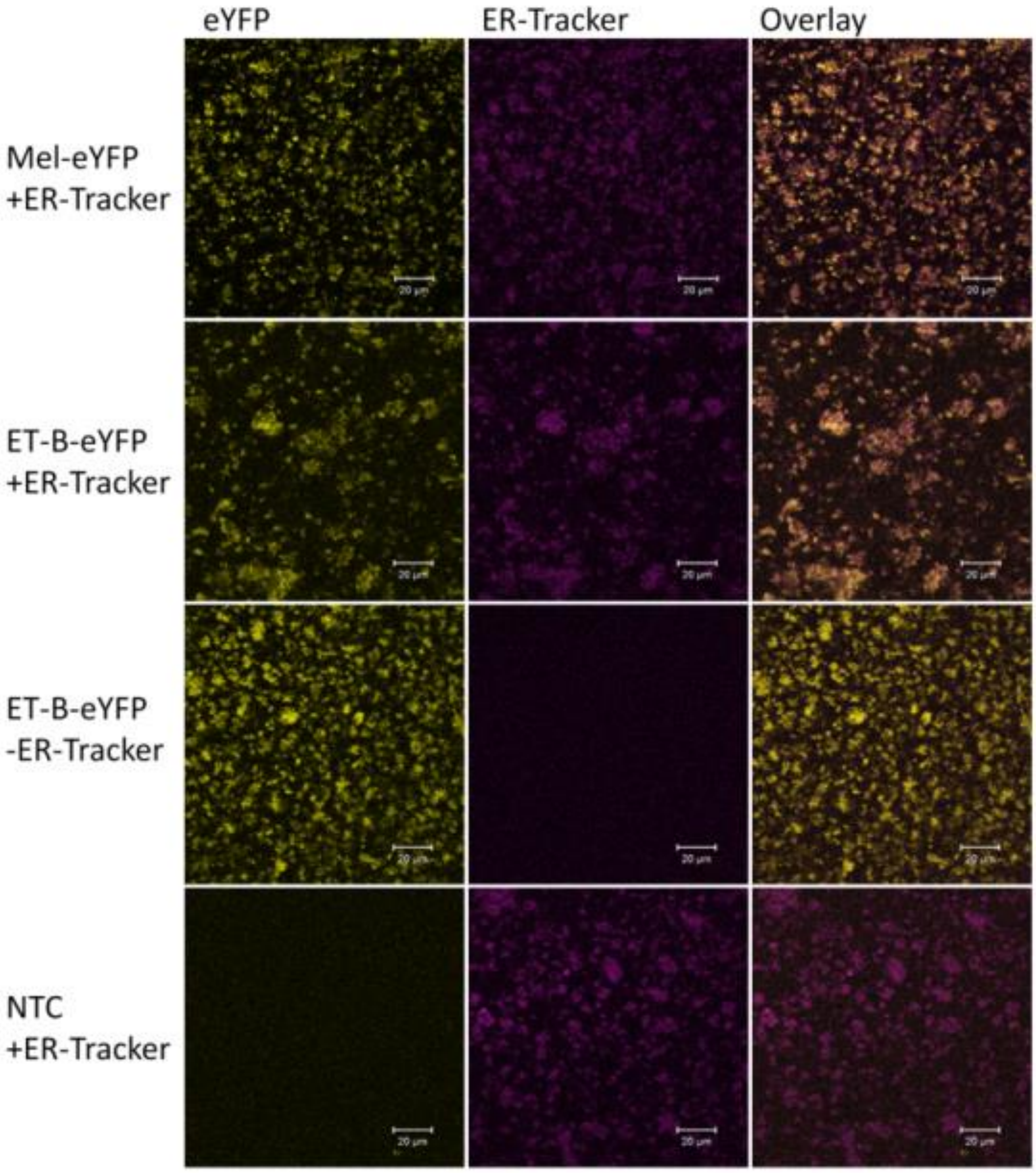

Supplementary figure 1: Confocal images displaying the co-localization of cell-free synthesized target proteins and molecular ER-Tracker in the microsomal fraction. The secretory Mel-eYFP and transmembrane protein ET-B were synthesized in a cell-free reaction and microsomal structures were subsequently labelled with the ER-Tracker probe. Microsomes were treated equally in the presence (+ER-Tracker) or absence of the Tracker (-ER-Tracker). NTC: no-template control

Supplementary Table 2. **Effect of defined Brij35 concentrations on the localization of the cytosolic protein Mel-Glyco-eYFP-His and the membrane protein ET-B-eYFP.** Percentage of proteins in the supernatant was calculated in relation to the total fluorescence signal in supernatant and vesicular fraction.

Concentrations of Brij35 (%)	Percentage of proteins in the supernatant (%)	
	Mel-Glyco-eYFP-His	ET-B-eYFP
0	41.5	16.6
0.01	40.3	23.5
0.02	77.6	25.4
0.03	84.6	41.5
0.04	85.9	55.6
0.05	85.7	58.0
0.06	87.9	59.1
0.07	88.1	61.2
0.08	87.8	62.2
0.09	87.7	62.6
0.1	87.7	62.5

Supplementary Table 3: Binding counts of [^{125}I]-ET-1 to ET-B receptor and negative control. The amount of radioactive labeled [^{125}I]-ET-1 bound to ET-B receptor and to a control containing no ET-B receptor in duplicate. Non-specific binding was subtracted from total binding to obtain specific binding.

TOTAL BINDING ET-B receptor

ET-1 [pM]	1	2	1	2	1	2	1	2	1	2	1	2	1	2
0,5	34	130,5	137	116,1	115,2	55,4	57,8	22,5	57,1	42,4	104,7	7,3	162,7	121,9
1	31,2	70,9	26,3	181,8	115,3	109,6	35,6	5,2	104,5	9	145	8,6	158,5	180
2	98,1	98,6	249,6	247,6	18,7	230,6	104,5	47,2	204	50,3	194	218,1	239,6	182,7
5	140,1	177,4	240,8	323,5	272,5	295,4	146,9	130,9	7,6	262,6	345,4	442,3	716,7	585,5
10	291,6	74,4	299,3	354,6	387,4	398,4	208,3	165,9	564,7	14,3	741,7	983,5	397	1124,6
20	300,1	91,4	353,1	422	512,6	471	121,2	315,3	415,3	59,7	862,8	32,5	1216,1	1373,4
50	879	794,3	916,5	766,8	860,5	916,4	725,9	517,4	792,1	804,7	15,7	1620,1	1972,5	1697,5
100	1269,3	1243,5	1354,4	1216	1440,7	1365,5	768,8	877,2	926,6	2129,4	2701,6	3331,3	75,6	2641
200	1488,8	2178	2791,2	2946,4	2728	2535,4	1730,2	1646,4	3395,8	6382,4	5584,6	4407,2	5461	5389,8

NON SPECIFIC BINDING ET-B receptor

ET-1 [pM]	1	2	1	2	1	2	1	2	1	2	1	2	1	2
0,5	103	111,4	157,5	128,8	91,5	115,6	19,2	27,5	27,1	20,1	187,8	94,9	74,3	45,7
1	56,1	676,8	130,6	683,7	102,5	137,4	24,3	30,8	7,5	29,3	112,7	148,2	36,1	88,4
2	47,1	40,3	24,8	137,7	148,9	207,1	42,4	61,9	68,7	86,9	238,9	185,6	111,1	99
5	54,5	87,4	179,1	155,7	204,2	196,5	69	57,55	170,9	88,25	471,2	425,4	333,3	306,7
10	156,9	100,5	272,1	230,9	268,4	214,6	150,5	215,7	338,4	369,4	930,5	766,9	647,4	505,3
20	183,1	206,1	302,7	308,2	333,1	401,3	181,5	166,85	258,8	253,2	345,5	354,5	660,7	531,6
50	507,9	401,3	484,6	553	568	662,2	393,3	363	441,8	525,45	1257,1	1329,8	1123,9	983,3
100	894,8	818,6	955	1155,7	1000,4	1070	875,4	847,4	48,4	907,5	2556,6	3089,3	24,6	1983,1
200	1689	1846,4	1802,8	2417	2014,2	2007	1387,2	1398,9	1940,6	114,4	101,4	4459	5343,2	3468,4

SPECIFIC BINDING (Total Binding - Non-Specific Binding) ET-B receptor

ET-1 [pM]	1	2	1	2	1	2	1	2	1	2	1	2	1	2
0,5	-69	19,1	-20,5	-12,7	23,7	-60,2	38,6	-5	30	22,3	-83,1	-87,6	88,4	76,2
1	-24,9				12,8	-27,8	11,3	-25,6	97	-20,3	32,3		122,4	91,6
2	51	58,3	224,8	109,9	-130,2	23,5	62,1	-14,7	135,3	-36,6	-44,9	32,5	128,5	83,7
5	85,6	90	61,7	167,8	68,3	98,9	77,9	73,35	-163,3	174,35	-125,8	16,9	383,4	278,8
10	134,7	-26,1	27,2	123,7	119	183,8	57,8	-49,8	226,3		-188,8	216,6		619,3
20	117		50,4	113,8	179,5	69,7	-60,3	148,45	156,5		517,3		555,4	841,8
50	371,1	393	431,9	213,8	292,5	254,2	332,6	154,4	350,3	279,25	-1241,4	290,3	848,6	714,2
100	374,5	424,9	399,4	60,3	440,3	295,5		29,8	878,2	1221,9	145	242		657,9
200		331,6	988,4	529,4	713,8	528,4	343	247,5	1455,2					

TOTAL BINDING negative Control

ET-1 [pM]	1	2	1	2	1	2	1	2	1	2	1	2
0,5	65,1	70,1	27,4	36,4	79,4	57,9	20,5	16,9	37,6	39,0	31,0	10,1
1,0	33,7	25,7	79,3	44,7	25,6	35,2	23,6	32,3	78,9	44,2	75,0	36,2
2,0	44,5	52,7	109,6	98,2	187,1	142,7	59,7	63,9	134,6	9,6	71,4	40,1
5,0	60,0	78,4	192,4	101,5	275,6	188,6	68,4	131,1	103,5	138,4	234,8	311,0
10,0	106,0	82,1	272,1	218,4	521,2	369,8	128,1	194,4	8,7	309,6	421,8	534,8
20,0	139,9	161,4	390,4	337,0	549,8	443,4	167,7	267,5	430,5	403,9	585,2	527,6
50,0	275,4	367,0	822,8	723,6	67,5	395,6	429,4	417,3	1595,8	1114,3	1087,9	1013,9
100,0	578,9	519,0	1486,3	1081,8	1228,6	1155,2	1281,5	1052,4	1529,4	1755,0	1675,0	2471,7
200,0	1396,0	1054,4	2354,0	2358,0	2660,4	1429,0	1541,2	1316,2	1940,6	114,4	3488,2	3228,2

NONSPECIFIC BINDING negative Control

ET-1 [pM]	1	2	1	2	1	2	1	2	1	2	1	2
0,5	87,4	83,4	18,3	18,4	94,4	8,6	15,4	23,0	24,4	14,3	23,1	9,4
1,0	46,2	32,8	140,5	25,0	112,3	116,1	36,9	28,3	43,3	51,2	46,6	39,9
2,0	51,7	57,7	26,6	170,3	273,1	201,1	41,2	57,2	78,2	73,4	69,2	51,5
5,0	81,8	84,6	234,7	177,5	208,4	216,2	53,2	61,8	115,1	95,3	210,0	322,9
10,0	9,9	146,4	332,6	275,1	287,6	249,0	154,4	137,1	247,1	227,0	615,0	410,6
20,0	179,3	206,9	460,6	382,0	342,0	392,8	150,2	179,9	301,0	441,0	550,6	556,2
50,0	347,1	432,8	671,6	863,7	666,4	729,3	392,5	371,6	637,4	1025,9	1007,5	1042,5
100,0	702,2	740,2	1316,0	1578,0	1066,6	1188,0	801,6	1058,4	44,5	1415,5	1833,9	1831,8
200,0	1235,4	1400,0	196,4	2830,8	2455,8	2160,6	1425,4	823,6	2015,0	2214,0	3680,6	3228,6

SPECIFIC BINDING (Total Binding - Non-Specific Binding) negative Control

ET-1 [pM]	1	2	1	2	1	2	1	2	1	2	1	2
0,5	-22,3	-13,3	9,1	18,0	-15,0	49,3	5,1	-6,1	13,2	24,7	7,9	0,7
1	-12,5	-7,1	-61,2	19,7	-86,7	-81,0	-13,3	4,0	35,6	-7,0	28,4	-3,7
2	-7,2	-5,0	83,0	-72,1	-86,0	-58,5	18,5	6,7	56,4	-63,8	2,2	-11,4
5	-21,8	-6,2	-42,3	-76,0	67,2	-27,7	15,2	69,3	-11,6	43,1	24,8	-11,9
10	96,1	-64,3	-60,5	-56,7	233,6	120,8	-26,3	57,3	-238,4	82,6	-193,2	124,2
20	-39,4	-45,5	-70,2	-45,0	207,8	50,6	17,5	87,6	129,5	-37,1	34,6	-28,6
50	-71,7	-65,8	151,2	-140,1	-598,9	-333,8	36,9	45,7	958,4	88,4	80,4	-28,6
100	-123,3	-221,2	170,3	-496,2	162,0	-32,8	479,9	-6,0	1484,9	339,5	-158,9	639,9
200	160,6	-345,6	2157,6	-472,8	204,6	-731,6	115,8	492,6	-74,4	-2099,6	-192,4	-0,4
